# Potential combinations of endocannabinoid/endocannabinoid-like compounds and antibiotics against methicillin-resistant *Staphylococcus aureus*

**DOI:** 10.1371/journal.pone.0231583

**Published:** 2020-04-15

**Authors:** Mark Feldman, Reem Smoum, Raphael Mechoulam, Doron Steinberg

**Affiliations:** 1 Biofilm Research Laboratory, Faculty of Dental Medicine, The Hebrew University of Jerusalem, Jerusalem, Israel; 2 The Institute for Drug Research, School of Pharmacy, The Hebrew University of Jerusalem, Jerusalem, Israel; National Institute of Animal Biotechnology, INDIA

## Abstract

Infections caused by antibiotic-resistant strains of *Staphylococcus aureus* have reached epidemic proportions globally. Our previous study showed antimicrobial effects of anandamide (AEA) and arachidonoyl serine (AraS) against methicillin (MET)-resistant *S*. *aureus* (MRSA) strains, proposing the therapeutic potential of these endocannabinoid/endocannabinoid-like (EC/EC-like) agents for the treatment of MRSA. Here, we investigated the potential synergism of combinations of AEA and AraS with different types of antibiotics against MRSA grown under planktonic growth or biofilm formation. The most effective combinations under planktonic conditions were mixtures of AEA and ampicillin (AMP), and of AraS and gentamicin (GEN). The combination with the highest synergy in the biofilm formation against all tested bacterial strains was AEA and MET. Moreover, the combination of AraS and MET synergistically caused default of biofilm formation. Slime production of MRSA was also dramatically impaired by AEA or AraS combined with MET. Our data suggest the novel potential activity of combinations of EC/EC-like agents and antibiotics in the prevention of MRSA biofilm formation.

## Introduction

The ability of bacterial pathogens to adapt and to overcome the challenges of antibiotics is a life-threatening problem that has emerged in the last few decades. Today, the increase in multidrug-resistant strains is a serious concern.

Despite the fact that *Staphylococcus aureus* are natural inhabitants of the human microbiota, severe staphylococcal infections can occur on epithelial surfaces [1], as well as in the bloodstream [2, 3]. *S*. *aureus* are very well adapted in the human body and extremely resistant to newly developed antibiotics with new targets of action. It has been postulated that these bacteria can develop resistance to any antibiotic [4]. Indeed, diseases associated with antibiotic-resistant strains of *S*. *aureus*, including methicillin (MET)-resistant *S*. *aureus* (MRSA), have spread globally [5] and are rapidly increasing in both healthcare and community settings [6–8]. In addition, the highly virulent community-associated MRSA strain causes tissue-destroying infections, such as necrotizing fasciitis and fulminant necrotizing pneumonia [9].

*S*. *aureus* can form biofilms on biotic and abiotic surfaces during infection. Very often, these biofilms are highly resistant to antimicrobials and are difficult to eradicate by host immune factors, since they act to protect bacteria from the effects of both antibiotics and the host immune system. Staphylococci in a biofilm environment have been shown to acquire heritable antibiotic resistance through spontaneous mutation [10], as well as plasmid-borne antibiotic resistance [11].

The EC system (ECS) is a biological system composed of EC, which are endogenous arachidonate-based lipids that bind to cannabinoid receptors, CB1 and CB2 that are expressed throughout both the central and peripheral nervous systems and peripheral organs. Enzymes in ECS are involved in synthesis and degradation of EC. CB1 and CB2 are activated by various substances such as EC or phytocannabinoids that occur naturally in the cannabis plant or synthetic cannabinoids [12]. The ECS is involved in the regulation of several physiological processes, including sleep and the immune response. Anandamide (AEA) is one of the main endogenous ligands of the cannabinoid receptors, recruited during tissue injury to provide a first response to nociceptive signals [13, 14]. Arachidonoyl serine (AraS), an EC-like lipid initially isolated from bovine brain, has been found to weakly bind to CB1 and CB2 receptors [15]. AraS demonstrates neuroprotection related to indirect signaling via the CB2 receptor [16]. Both agents contribute to the maintenance of vascular integrity and angiogenesis [17, 18]. Moreover, AEA has been shown to diminish the inflammatory response in periodontitis [19]. A few studies have reported the antimicrobial effects of cannabis extracts against different pathogens [20], and anti-MRSA activity of exogenous cannabinoids [21]. In addition, we have shown that single AEA and AraS effectively alter the pathogenicity of different MRSA strains [22].

In the present study, we investigated the potential synergistic effects of combining EC and EC-like compounds AEA and AraS with different antibiotics against MRSA growing under planktonic growth or biofilm formation.

## Materials and methods

### The tested compounds

**Fig 1:**
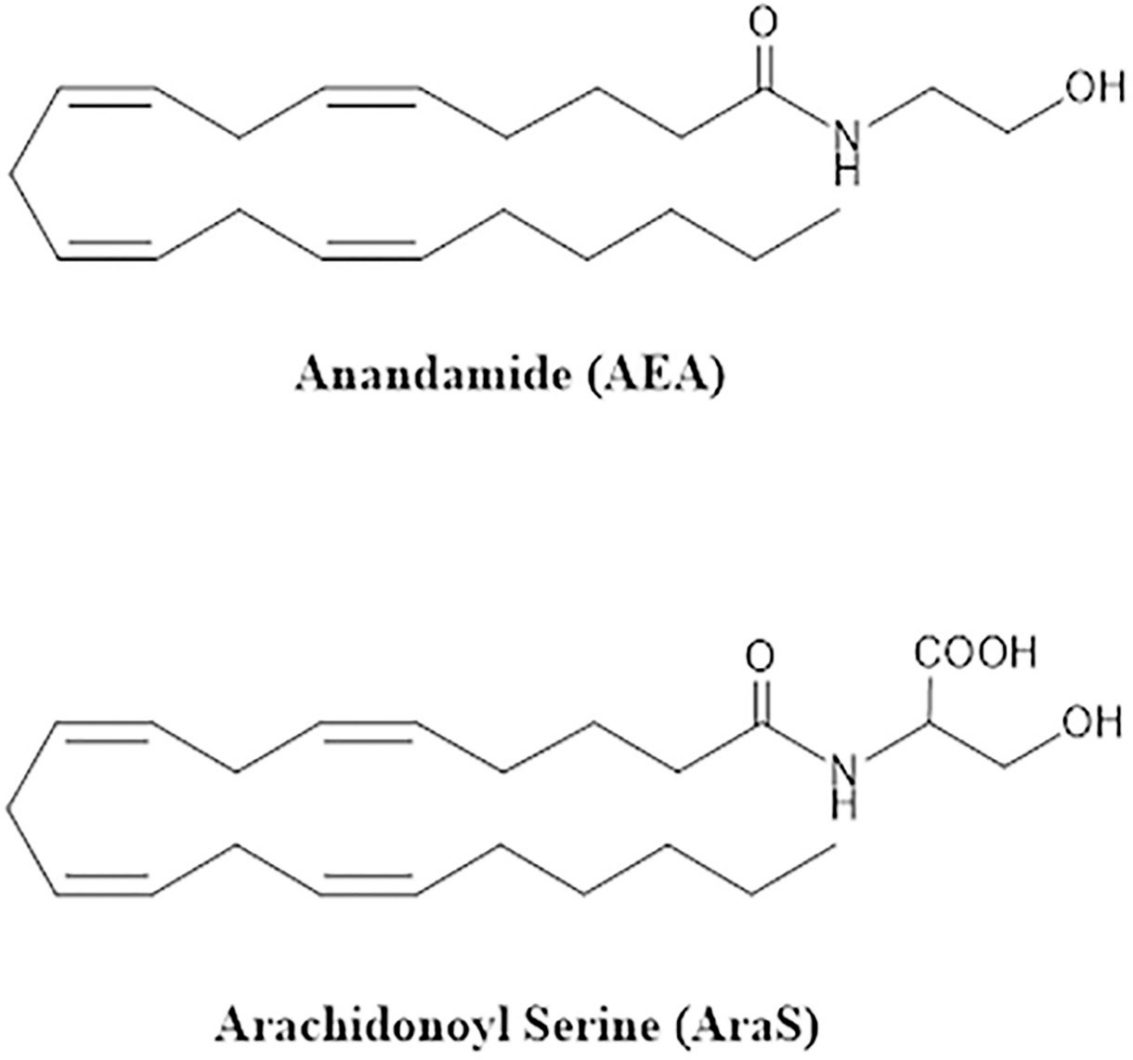
Structure of EC and EC-like compounds.

AEA was synthesized following the procedure described by Devane et al. [23]. AraS was prepared following the procedure described by Milman et al. [15] (Fig 1). The tested antibiotics were: ampicillin (AMP), gentamicin (GEN), methicillin (MET), and tetracycline (TET) (all from Sigma–Aldrich, St. Louis, MO).

### Preparation of bacterial inoculum

The following MRSA strains were used in this study: a clinical isolate (CI) obtained from Hadassah Medical Center, Jerusalem, Israel, ATCC 33592 (isolated from blood) and ATCC 43300 (clinical isolate). All of the bacterial strains were kept at -80°C and cultured and incubated from frozen stock in tryptic soy broth (TSB, Neogen, Lansing, MI) at 37°C for 24 h.

### Determination of minimal inhibitory concentration (MIC)

The ability of EC/EC-like compounds and the tested antibiotics to inhibit planktonic growth of MRSA strains was determined by a twofold serial microdilution method based on a CLSI protocol as described in our previous study [22]. Serial 1:2 dilutions of each tested EC/EC-like compound/antibiotic in BHI broth (from 4 μg/ml to 256 μg/ml) were prepared in a polystyrene flatbottomed 96-well microplate. Wells with no EC/EC-like compound/antibiotics and with bacteria served as positive controls. Wells with no bacteria and with EC/EC-like compound/antibiotics served as blanks. Then, an equal volume (100 μl) of the bacterial suspension at 0.5 McFarland turbidity was added. After a 24-h incubation at 37°C under aerobic conditions, supernatants containing free-floating bacteria from each well were transformed to the new plate and growth was monitored by recording the OD at 595 nm using a Genious plate reader Spectrophotometer (Tecan). Three independent experiments were performed in triplicate. Data are presented as mean ± SD.

### Determination of minimal biofilm inhibitory concentration (MBIC)

The ability of EC/EC-like compounds and tested antibiotics to inhibit biofilm formation of MRSA strains was determined by the method described in our previous study [22]. All tested EC/EC-like compound/antibiotics were applied at doses of 4 μg/ml–256 μg/ml. The assay was performed as described above except of growth medium used was TSB, supplemented with 1% glucose. After incubation for 24 h spent media and free-floating bacteria were removed by aspiration and the wells were washed twice with phosphate-buffered saline (PBS, pH 7.4), prior to quantify biofilm by crystal violet staining [28]. Briefly, 0.02% crystal violet was added into wells for 45 min, which were then washed twice with DDW to remove unbound dye. After adding 200 μl of 30% acetic acid into each well, the plate was shaken for 10 min to release the dye and the biofilm was quantified by measuring the absorbance at 595 nm using a Genious plate reader Spectrophotometer (Tecan). Three independent experiments were performed in triplicate. Data are presented as mean ± SD.

### Determination of fractional inhibitory concentration index (FICI) and fractional biofilm inhibitory concentration index (FBICI)

#### Checkerboard assay for planktonic bacteria

The stock solutions and serial twofold dilutions of each of the tested compounds (AEA and AraS) and of the tested antibiotic were prepared immediately prior to testing [24]. Briefly, AEA or AraS was serially diluted along the ordinate side of the microdilution plate, while the tested antibiotic was diluted along the abscissa. Bacterial inoculum at 0.5 McFarland turbidity was prepared from each of the tested bacterial strains. The result was a checkerboard of combinations of the two agents, with control wells of each of the agents. Incubation procedure and determination of planktonic growth were performed as described in **Determination of MIC** section. The FICI, based on fractional inhibitory concentration (FIC), was calculated as follows: FIC (A+B)/FIC A + FIC (B+A)/FIC B, where FIC (A+B) is the MIC of drug A in the combination/MIC of single drug A, and FIC B is the MIC of drug B in the combination/MIC of single drug B. The combination is considered synergistic when FICI ≤ 0.5, partially synergistic at 0.5 < FICI ≤ 1, indifferent at 1 < FICI ≤ 4, and antagonistic at FICI > 4 [25].

#### Checkerboard assay for biofilm

The assay was performed as described above in **Determination of MBIC** section. Biofilm biomass was quantified using crystal violet staining as described above. The FBICI, based on fractional biofilm inhibitory concentration (FBIC), was calculated as follows: FBIC (A+B)/FBIC A + FBIC (B+A)/FBIC B, where FBIC (A+B) is the MBIC of drug A in the combination/MBIC of single drug A, and FBIC B is the MBIC of drug B in the combination/MBIC of single drug B. The combination is considered synergistic when FBICI ≤ 0.5, partially synergistic at 0.5 < FBICI < 1, indifferent at 1 < FBICI < 4, and antagonistic at FBICI > 4 [25].

### Light microscopy analysis of formed biofilms

Biofilms of MRSA strain ATCC 43300 formed in the presence of 1/2 MBIC of single AraS and MET and 1/2 FBIC in combination were examined by light microscopy using an EVOS® FL Imaging System (Thermo Scientific, Waltham, MA). Biofilms were formed in a 96-well plate as described above. Untreated biofilm served as a control. Formed biofilms were visualized and photographed at 40X magnification. At least four random fields were observed. Three independent experiments were performed and one set of representative results is shown.

### Slime-production assay using Congo Red Agar (CRA)

Colony morphology and slime production were investigated using CRA, as previously described [26]. The CRA was composed of 37 mg/ml brain–heart infusion broth (BD Biosciences, Franklin Lakes, NJ), 36 mg/ml sucrose (Sigma), 15 mg/ml agar (BD Biosciences), and 0.8 mg/ml Congo red (Sigma). MRSA strains ATCC 33592 and ATCC 43300 were incubated at 1/2 MBIC of AraS/AEA and the antibiotic single MET, and 1/2 FBIC for their combinations in a 96-well plate for 24 h under the conditions described above. Treated and untreated controls were then drop-plated on CRA and incubated for 24 h at 37°C before taking images. Assays were performed in triplicate. Positive results were indicated by black colonies, whereas weak slime producers remained pink. Three independent experiments were performed and one set of representative results is shown.

## Results

### MIC

Using the standard broth microdilution methods, we evaluated the MICs of the tested agents. At the tested concentrations, the endocannabinoid AEA did not exhibit inhibitory effect on the growth of the tested MRSA strains at all tested doses (Tables 1–4). In contrast, the MICs of AraS varied with the different tested strains: 28.4 μg/ml for ATCC 33592, 128 μg/ml for ATCC 43300, and >256 μg/ml for CI (Tables 1–4). The bacteria were resistant to the tested antibiotics. Among the tested antibiotics, TET demonstrated the lowest MIC (32 μg/ml) against all MRSA strains (Table 4), whereas AMP (Table 1) and GEN (Table 2) were less potent (128 μg/ml ->256 μg/ml). The effect of MET on bacterial growth varied among the different strains: 32 μg/ml for ATCC 33592, 32 μg/ml for ATCC 43300 and >256 μg/ml for CI (Table 3).

**Table 1:** Effects of the combinations of AraS/AEA with AMP against MRSA strains.

MRSA strain	Agents	Planktonic growth				Biofilm formation			
		MIC, μg/ml	FIC, μg/ml	FICI	Effect	MBIC, μg/ml	FBIC, μg/ml	FBICI	Effect
CI	AraS	>256±0	>256±0	>1<4	indifferent	32±0	32±0	>1<4	indifferent
	AMP	256±0	256±0			256±0	64±0		
ATCC 33592	AraS	28.4 ±7.1	8±0	<0.5≤1	partial synergy	30.2±5.3	14.2±3.5	<0.5≤1	partial synergy
	AMP	128±0	64±0			128±0	32±0		
ATCC 43300	AraS	128±0	15.1±2.7	≤0.5	synergy	30.2±5.3	7.1±1.8	≤0.5	synergy
	AMP	256±0	16±0			256±0	64±0		
CI	AEA	>256±0	28.4±7.1	≤0.5	synergy	30.2±5.3	16±0	<0.5≤1	partial synergy
	AMP	256±0	16±0			256±0	64±0		
ATCC 33592	AEA	>256±0	8±3.5	≤0.5	synergy	33.8±12.5	8±0	≤0.5	synergy
	AMP	128±0	8±0			128±0	8±0		
ATCC 43300	AEA	>256±0	16.9±6.3	≤0.5	synergy	>256±0	15.1±2.7	≤0.5	synergy
	AMP	256±0	8±0			256±0	8±0		

Data are presented as means ± SD of three independent experiments performed in triplicate (n = 9).

**Table 2:** Effects of the combinations of AraS/AEA with GEN against MRSA strains.

MRSA strain	Agents	Planktonic growth				Biofilm formation			
		MIC, μg/ml	FIC, μg/ml	FICI	Effect	MBIC, μg/ml	FBIC, μg/ml	FBICI	Effect
CI	AraS	>256±0	33.8±12.5	≤0.5	synergy	32±0	16±0	<0.5≤1	partial synergy
	GEN	256±0	32±0			256±0	64±0		
ATCC 33592	AraS	28.4 ±7.1	4.2±1.6	≤0.5	synergy	30.2±5.3	4.9±1.8	≤0.5	synergy
	GEN	128±0	4±0			128±0	4±0		
ATCC 43300	AraS	128±0	30.2±5.3	≤0.5	synergy	30.2±5.3	7.1±1.8	≤0.5	synergy
	GEN	256±0	16±0			256±0	64±0		
CI	AEA	>256±0	>256±0	>1<4	indifferent	30.2±5.3	16.9±6.3	<0.5≤1	partial synergy
	GEN	256±0	256±0			256±0	32±0		
ATCC 33592	AEA	>256±0	7.6 ±1.3	≤0.5	synergy	33.8±12.5	8,4±3.1	≤0.5	synergy
	GEN	128±0	4±0			128±0	8±0		
ATCC 43300	AEA	>256±0	>256±0	>1<4	indifferent	>256±0	14.2±3.5	≤0.5	synergy
	GEN	256±0	256±0			256±0	32±0		

Data are presented as means ± SD of three independent experiments performed in triplicate (n = 9).

**Table 3:** Effects of the combinations of AraS/AEA with MET against MRSA strains.

MRSA strain	Agents	Planktonic growth				Biofilm formation			
		MIC, μg/ml	FIC, μg/ml	FICI	Effect	MBIC, μg/ml	FBIC, μg/ml	FBICI	Effect
CI	AraS	>256±0	>256±0	>1<4	indifferent	32±0	16±0	<0.5≤1	partial synergy
	MET	>256±0	>256±0			>256±0	16±0		
ATCC 33592	AraS	28.4 ±7.1	7.1±1.8	≤0.5	synergy	30.2±5.3	15.1± 2.7	<0.5≤1	partial synergy
	MET	32±0	8±0			32±0	8±0		
ATCC 43300	AraS	128±0	15.1± 2.7	≤0.5	synergy	30.2±5.3	7.6± 1.3	≤0.5	synergy
	MET	32±0	2±0			32±0	8±0		
CI	AEA	>256±0	14.2±3.5	≤0.5	synergy	30.2±5.3	8±3.5	≤0.5	synergy
	MET	>256±0	16±0			>256±0	16±0		
ATCC 33592	AEA	>256±0	16.9± 6.3	<0.5≤1	partial synergy	33.8±12.5	7.1±1.8	≤0.5	synergy
	MET	32±0	16±0			32±0	8±0		
ATCC 43300	AEA	>256±0	16.9±6.3	≤0.5	synergy	>256±0	28.4±7.1	≤0.5	synergy
	MET	32±0	8±0			32±0	8±0		

Data are presented as means ± SD of three independent experiments performed in triplicate (n = 9).

**Table 4:** Effects of the combinations of AraS/AEA with TET against MRSA strains.

MRSA strain	Agents	Planktonic growth				Biofilm formation			
		MIC, μg/ml	FIC, μg/ml	FICI	Effect	MBIC, μg/ml	FBIC, μg/ml	FBICI	Effect
CI	AraS	>256±0	30.2±5.3	<0.5≤1	partial synergy	32±0	16±0	<0.5≤1	partial synergy
	TET	32±0	16±0			32±0	16±0		
ATCC 33592	AraS	28.4±7.1	28.4±7.1	>1<4	indifferent	30.2±5.3	33.8±12.5	>1<4	indifferent
	TET	32±0	2±0			32±0	2±0		
ATCC 43300	AraS	128±0	32±0	<0.5≤1	partial synergy	30.2±5.3	16.9±6.3	<0.5≤1	partial synergy
	TET	32±0	16±0			32±0	2±0		
CI	AEA	>256±0	7.6±1.3	<0.5≤1	partial synergy	30.2±5.3	15.1±2.7	<0.5≤1	partial synergy
	TET	32±0	16±0			32±0	16±0		
ATCC 33592	AEA	>256±0	15.1±2.7	<0.5≤1	partial synergy	33.8±12.5	15.1±2.7	<0.5≤1	partial synergy
	TET	32±0	16±0			32±0	16±0		
ATCC 43300	AEA	>256±0	8±3.5	<0.5≤1	partial synergy	>256±0	14.2±3.5	≤0.5	synergy
	TET	32±0	16±0			32±0	1±0		

Data are presented as means ± SD of three independent experiments performed in triplicate (n = 9).

### MBIC

AEA inhibited biofilm formation of two MRSA strains, CI and 33592, at a dose of approximately 32 μg/ml which was more than 8-fold lower than its MIC (>256 μg/ml) against these strains (Tables 1–4). Biofilm formation of MRSA strain 43300 was not affected by AEA (Tables 1–4). MBIC of AraS was the same or less than its MIC against the respective strain (Tables 1–4). The MBIC values of all tested antibiotics were the same as their MIC values (Tables 1–4).

### Evaluation of combined antibacterial effect

#### Planktonic growth

The most effective combinations under planktonic growth were the mixtures of AEA and AMP (Table 1), and of AraS and GEN (Table 2). These mixtures showed synergistic effects against all tested MRSA strains. For instance, the combination of AEA and AMP against MRSA strain ATCC 33592 (FIC = 8 μg/ml for both, AEA and AMP) dramatically reduced the MIC of AEA (>256 μg/ml) by more than 32-fold, and the MIC of AMP (128 μg/ml) by 16-fold (Table 1). The combination of AraS and GEN (FIC = 4 μg/ml and 4.2 μg/ml for GEN and AraS, respectively) also demonstrated a notable synergistic effect against MRSA strain ATCC 33592, where the MIC of AraS (28.4 μg/ml) was decreased by 8-fold, while that of GEN (128 μg/ml) was decreased by 32-fold (Table 2).

#### Biofilm formation

The most potent synergistic combination in the biofilm formation against all tested bacterial strains was the mixture of AEA and MET (Table 3). For instance, the combination of AEA and MET against MRSA strain CI (FBIC = 8 μg/ml for AEA and 16 μg/ml for MET) reduced the MBIC of AEA (30.2 μg/ml) by about 4-fold, and the MBIC of MET (>256 μg/ml) by 16-fold (Table 3). Among the three tested antibiotics, the best combination with AraS was with GEN: A synergistic effect was observed in the biofilm formation against MRSA strains ATCC 33592 and ATCC 43300, and partial synergy was seen against the MRSA CI (Table 2). For instance, the combination of AraS and GEN against MRSA strains ATCC 33592 (FBIC = 4.9 μg/ml for AraS and 4 μg/ml for GEN) reduced the MBIC of AraS (30.2 μg/ml) by about 6-fold, and the MBIC of GEN (128 μg/ml) by 32-fold (Table 2). Moreover, the combination of AEA with antibiotics was more potent than that of AraS, with the respective antibiotics, against MRSA biofilm formation. Combinations of AEA and antibiotics demonstrated mostly synergistic effects on biofilm formation, whereas combinations of AraS and antibiotics in the biofilm condition were mostly partially synergistic, or indifferent (Tables 1–4).

### Biofilm structure

**Fig 2:**
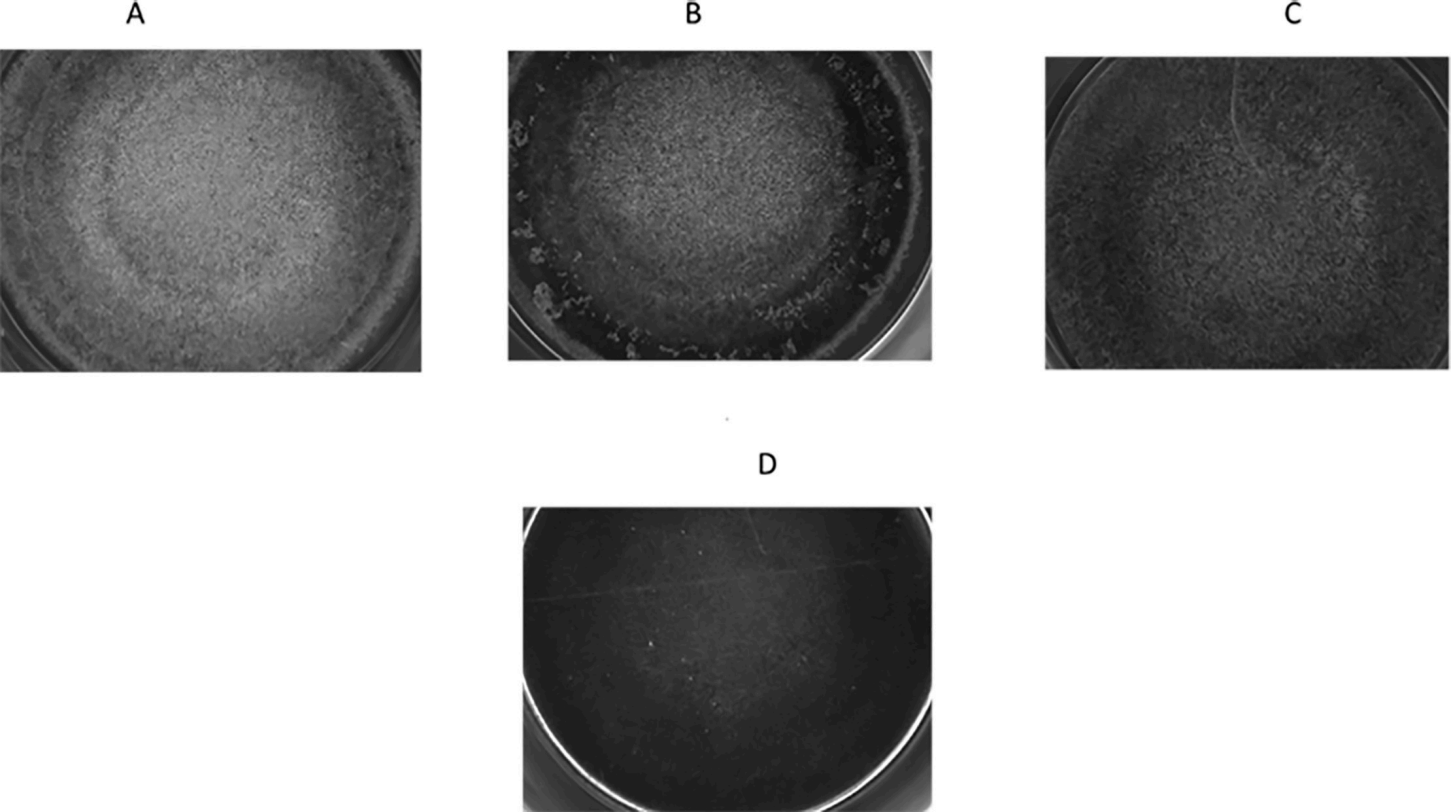
Structure of biofilms. Light microscopy images of formed biofilms. (A) Untreated. (B) Treated with single AraS. (C) Treated with single MET. (D) Treated with the combination of AraS and MET.

We visualized the inhibitory effects of single AraS and MET or in combination, on biofilm formation of MRSA strain ATCC 43300, by light microscopy. Each agent, when administered as single at 1/2 MBIC (16 μg/ml of each agent), reduced the biomass and altered the structure of the biofilm in different manners compared to the untreated control. Control biofilm showed an intact biomass homogeneously spread around the well (Fig 2A). Single AraS notably reduced biomass at the periphery of the well, while minory affecting the formed biofilm at the center of the well (Fig 2B). In contrast, single MET was able to decrease biomass across the well, but still allowed biofilm formation (Fig 2C). However, when MRSA biofilm was exposed to the combination of AraS and MET, each at 1/2 FBIC (4 μg/ml of each agent) (Table 3), biofilm formation was almost totally inhibited (Fig 2D).

### Slime production

We determined the effect of the compounds applied as single or in combination on biofilm-associated slime production of MRSA strains ATCC 33592 and ATCC 43300.

#### MRSA ATCC 33592

**Fig 3:**
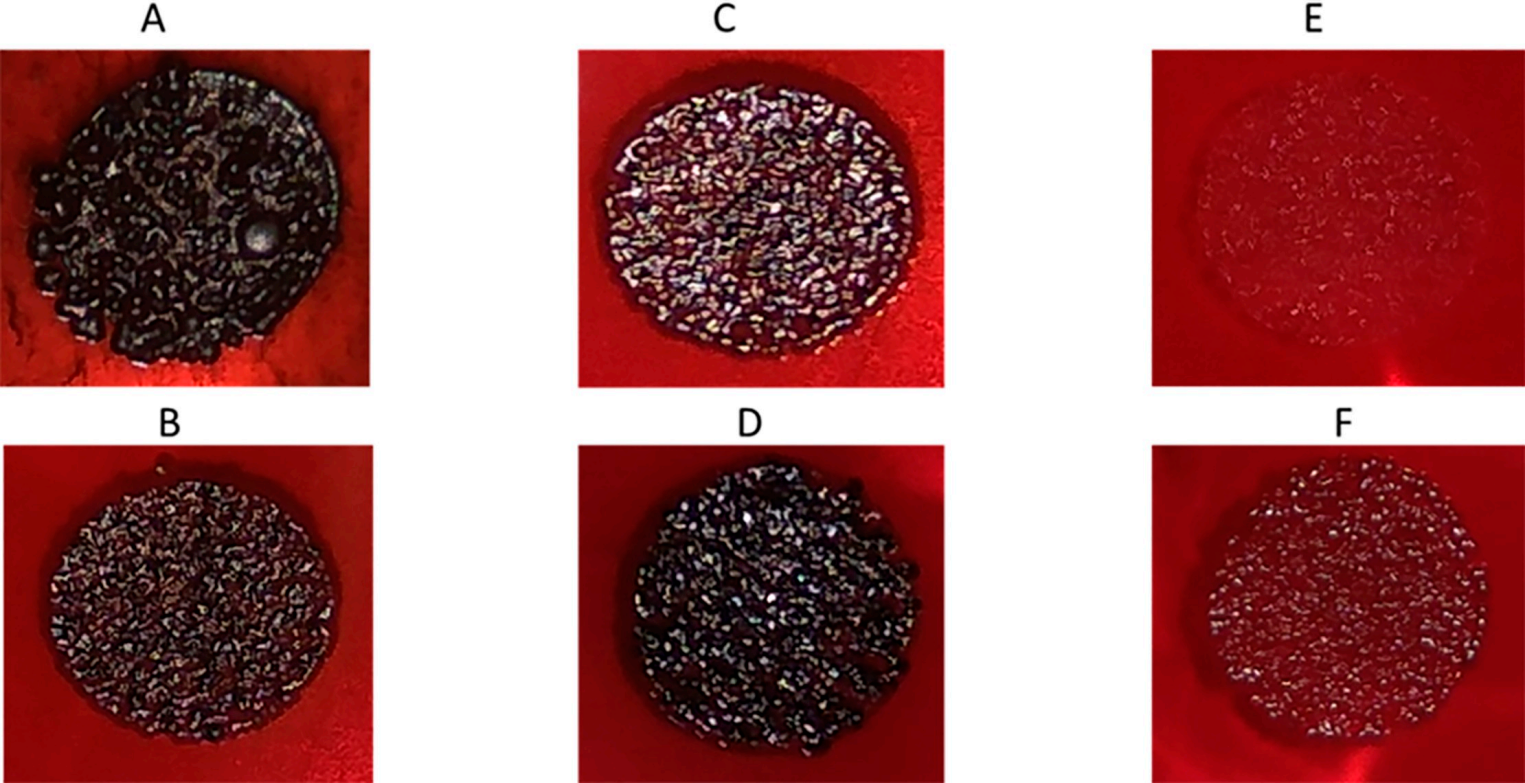
Slime production of MRSA strain 33592. Slime production was observed using CRA plates. (A) Untreated. (B) Treated with single MET. (C) Treated with single AEA. (D) Treated with single AraS. (E) Treated with a combination of AEA and MET. (F) Treated with a combination of AraS and MET.

MRSA ATCC 33592 is a high slime-producing strain, appearing as black colonies on CRA (Fig 3A). Moreover, the surface of the colonies show high roughness (Fig 3A). Single MET (Fig 3B) and AEA (Fig 3C), each at 1/2 MBIC (16 μg/ml of each agent), were able to slightly reduce the roughness and black colour of the colonies, whereas single AraS at 1/2 MBIC (16 μg/ml) had almost no effect (Fig 3D). However, when combinations at 1/2 FBIC of MET (4 μg/ml) with 1/2 FBIC of AEA (4 μg/ml) (Fig 3E) or AraS (8 μg/ml) (Fig 3F) were applied, colony colour and morphology changed drastically. Both combinations totally eliminated the black colour and reduced roughness, resulting in red colonies with a flat structure (Fig 3E and 3F), indicating strong alteration of slime production.

#### MRSA ATCC 43300

**Fig 4:**
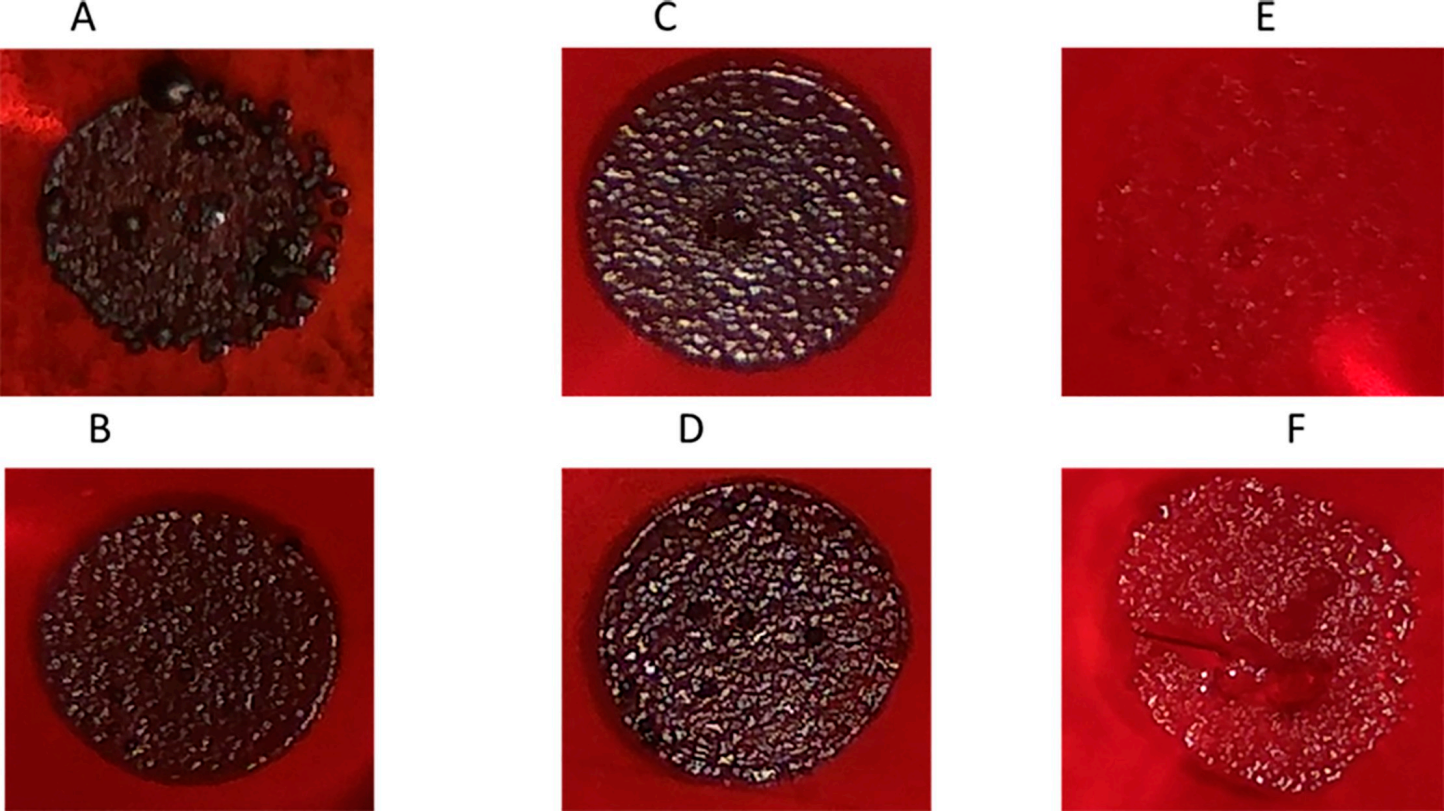
Slime production of MRSA strain 43300. Slime production was observed using CRA plates. (A) Untreated. (B) Treated with single MET. (C) Treated with single AEA. (D) Treated with single AraS. (E) Treated with a combination of AEA and MET. (F) Treated with a combination of AraS and MET.

Similar to MRSA strain ATCC 33592, untreated MRSA ATCC 43300 appeared as black rough colonies on CRA (Fig 4A). All tested agents, MET (Fig 4B), AEA (Fig 4C) and AraS (Fig 4D), applied as single at 1/2 MBIC (16 μg/ml, 128 μg/ml and 15.1 μg/ml, respectively) slightly reduced roughness, but barely affected slime production. However, when biofilm was exposed to combinations of MET at 1/2 FBIC (4 μg/ml) with 1/2 FBIC of AEA (14.2 μg/ml) (Fig 4E) or AraS (3.8 μg/ml) (Fig 4F), colony color and morphology changed visibly. As observed for MRSA strain ATCC 33592, black colonies with high roughness were dramatically modified to red colonies with flat structure (Fig 4E and 4F). As mentioned above for MRSA strain ATCC 33592, this effect was associated with a notable reduction in bacterial slime production.

## Discussion

Bacterial infections, especially those associated with resistant bacteria, constitute a clinical problem. A novel suggested approach to overcoming bacterial resistance to antibiotics is the use of a wide range of alternative antimicrobial compounds that act synergistically with known antibiotics [27]. Multidrug resistant bacteria is more difficult to treat than bacteria which are resistant to a single antibiotic, and MRSA strains call for special attention, since they have developed resistance to different classes of antibiotics [28, 29]. Indeed, in our study, all tested MRSA strains, whether in a planktonic environment or in biofilm, were highly resistant to three classes of antibiotics: beta-lactams (AMP and MET), aminoglycoside (GEN) and TET.

Antimicrobial combinations are aimed at obtaining a synergistic interaction in which one compound elevates the individual activity of another compound in combination and *vice versa* [30]. For instance, anti-biofilm compounds have been shown to act synergistically with antibiotics and improve their inhibitory action against chronic infections [31]. Here, we demonstrate that combinations of EC/EC-like compounds with antibiotics effectively reduce the MIC and MBIC of each of the single agents, thus demonstrating a synergistic impact. Antibiotics stop the growth of bacteria and thus prevent the biofilm formation by non-specific bactericidal action. In contrast, the tested EC/EC-like compounds, especially AEA, exhibited specific non-killing anti-biofilm activity, in accordance with our previous study. This agent had no detectable MIC, but notably reduced biofilm formation of all tested MRSA strains. Biofilm adaptation among microbes is one of the leading causes of antibiotic resistance [22]. We demonstrated that a combination of AEA or AraS with antibiotics exhibits much higher anti-biofilm activity than each of these single agents. Similarly, hamamelitannin, a quorum-sensing (QS) inhibitor, in conjunction with antibiotics such as vancomycin or clindamycin, was effective at killing *S*. *aureus* biofilm cells at levels greater than single QS inhibitor or antibiotic [32].

*S*. *aureus* produces the biofilm-associated polysaccharide glycocalyx, known as slime, which is involved in adherence to and colonization of biomedical devices. Slime formation in MRSA strains is associated with severe and even fatal infection [33]. Interestingly, slime production was barely affected at 1/2 MBIC of each agent tested in our study when applied as single. However, mixtures of EC/EC-like compounds and antibiotics, even at 1/8 MBIC, dramatically attenuated staphylococcal slime production. Therefore, we postulated that inhibition of slime production by the combination of EC/EC-like compounds and antibiotics might reduce the virulence of MRSA.

Both AEA and AraS are derivatives of arachidonic fatty acid. Fatty acids have been shown to act synergistically with antibiotics against *S*. *aureus* strains [34, 35]. Among the agents tested in this study, AEA exhibited a more pronounced synergistic effect against MRSA immobilized in biofilm than AraS at non-killing concentrations.

ECs are amphiphilic molecules that have been shown to interact with mammalian cell membrane via a non-specific receptor-independent mechanism [36]. It has been proposed that ECs can modify lipid bilayers’ fluidity [37, 38] and elastic properties [36]. Since ECs modify the eukaryotic membrane lipid bilayer non-specifically, we assume that these amphiphilic compounds can act similarly on the prokaryotic cell membrane lipid bilayer and therefore, when combined with antibiotics, they can enhance permeability to, and efficiency of antibiotics. Related observations have been published. For instance, the amphiphilic peptide magainin II demonstrated synergy in combination with beta-lactam antibiotics due to its increased access to the bacterial (including 30 MRSA strains) cytoplasmic membrane, following the breakdown of peptidoglycan by the beta-lactam antibiotics [39]. Another study demonstrated that the amphiphilic biosurfactant sophorolipid interacts positively with TET against *S*. *aureus* via alteration of bacterial membrane integrity, which facilitates penetration of the antibiotic [40]. Furthermore, the amphiphilic nanocarrier C1-PNC compromises the MRSA cell membrane; combined with GEN, this allows elevated cellular uptake of the antibiotic, which in turn leads to elimination of bacterial cells [41]. Our previous results demonstrated that single AEA or AraS, at sub-killing concentrations, were able to destabilize the cytoplasmic membrane of MRSA [22].

Accordingly, we propose that one of the modes of the synergistic interaction between EC/EC-like compounds and antibiotics against MRSA growth and biofilm formation is enhancement of the uptake and the bactericidal/anti-biofilm efficiency of the therapeutic antibiotic by the non-specific membrane-targeting activity of the compounds. In addition, the reduction in slime production probably enhances accessibility of the agents to the bacterial membrane.

Another possible mode of this effective interaction could be attributed to the previously reported anti-QS activity of AEA [42]. In that study, we demonstrated that AEA acts as a structure-specific QS inhibitor in the Gram-negative bacteria *Vibrio harveyi* [42]. QS is strongly associated with enhanced drug-resistance of biofilms [43] and, therefore, interfering with the bacterial communication system could be a promising strategy to combat resistant pathogens. Moreover, as already noted, a QS inhibitor can exhibit synergistic interactions with antibiotics against *S*. *aureus* [32]. However, further research is required to investigate the anti-QS effect of AEA toward MRSA.

The important outcome of this combination is interruption of the core resistance mechanism in MRSA. In addition, the synergistic combination of the ECs and antibiotics not only enhanced the anti-MRSA efficacy of the antibiotics but also lowering their effective dose, thereby reducing the risk of antibiotic-associated side effects in the host.

In conclusion, our data show a synergistic interaction between EC/EC-like compounds and antibiotics against MRSA growth and biofilm formation. As such, they reveal a further biological role for these endogenous agents, as a potential line of defense for the host against bacterial invasion.
